# Extreme Food-Plant Specialisation in *Megabombus* Bumblebees as a Product of Long Tongues Combined with Short Nesting Seasons

**DOI:** 10.1371/journal.pone.0132358

**Published:** 2015-08-12

**Authors:** Jiaxing Huang, Jiandong An, Jie Wu, Paul H. Williams

**Affiliations:** 1 Key Laboratory for Insect-Pollinator Biology of the Ministry of Agriculture, Institute of Apicultural Research, Chinese Academy of Agricultural Sciences, Beijing 100093, China; 2 Department of Life Sciences, The Natural History Museum, London SW7 5BD, United Kingdom; Institute of Zoology, CHINA

## Abstract

*Megabombus* bumblebees have unusually long tongues and are generally more specialised than other bumblebees in their choice of food plants. The phylogeny of *Megabombus* bumblebees shows that speciation was concentrated in two periods. Speciation in the first period (*ca* 4.25–1.5 Ma) is associated with the late rise of the Hengduan Mountains at the eastern end of the Qinghai-Tibetan plateau. Speciation in the second period (1.2–0.3 Ma) is associated with climatic cooling in the northern forests. The most extreme food-specialist species belong to the second period, which may point to climate as a factor in specialisation. These extreme specialist species occur either in the far north (*Bombus consobrinus*), or at high elevations (*Bombus gerstaeckeri*), in situations where long tongues coincide with the shortest nesting seasons. Species with the longest tongues but occurring further south (even at high elevations) use a broader range of food plants.

## Introduction

Bumblebees are important pollinators for wild plants and crops. Bumblebee species with longer tongues are more specialised in their choice of food plants than with shorter-tongued species for both nectar and pollen [1,2,3,4]. This greater specialisation has even been considered as a factor contributing to the greater susceptibility of some long-tongued bumblebees to population declines [5,6,7]. Narrower dietary breadth is suggested to be associated with longer proboscides, which have a major influence on the adaption of habitat, affect the population decline [8,9]. Therefore, understanding the evolution of longer tongue and dietary breadth among the long tongue bee is key to develop an effective conservation strategies for these food specialization bumblebees. But in order to study the evolution of specialization, we need to recognise species and understand the phylogeny of these long tongue bumblebees.

Among bumblebees, the subgenus *Megabombus* (in the broad sense of Williams et al. [10]) is a group that includes many of the longest-tongued and most specialised bumblebee species in the world [4,10,11]. It becomes one of the excellent group for exploring the tongue length and food specialization evolution. For species recognizing in *Megabombus*, Skorikov recognised two subgenera *Hortobombus* and *Diversobombus*, in which together he estimated 15 species [12]. While recent survey by Williams suggested that there are 22 species including three subgenus *Diverobombus*, *Megabombus* and *Senexibombus*[13]. Within those long tongue bumblebees, there are at least two extreme specialists that visit only one food-plant species for pollen.

**Fig 1:**
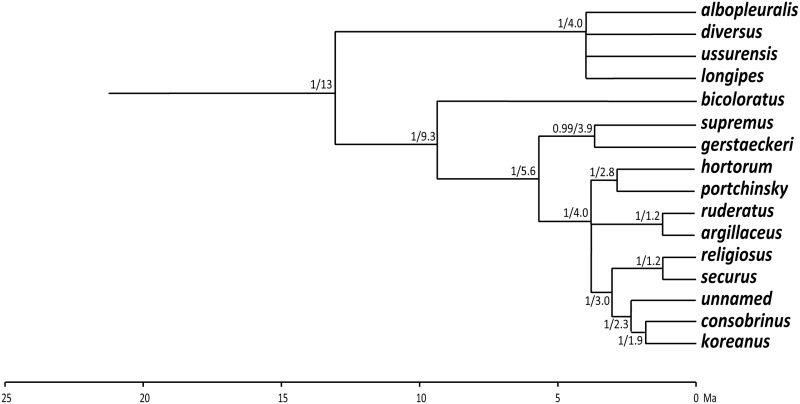
Earlier estimate of phylogeny for species of the subgenus *Megabombus*. From combined Bayesian analysis of five genes: (mitochondrial) 16S, and (nuclear) opsin, ArgK, EF-1α, and PEPCK [11]. Values at each node: posterior probability / age in Ma. Redrawn with nodes with support *p*<0.66 shown collapsed. Date estimates in millions of years before the present (Ma) are taken from Hines (her Fig 2) [33]. Species concepts and names are adjusted according to the interpretations of the present study.

*Megabombus* has been claimed to be more diverse in some of the mountains of China [13,14,15] compared with Europe [13,16]. But is this true? The *Megabombus* species of China are still poorly understood [11,17]. There is just one previous molecular study of almost all bumblebees [11], which included an estimate of phylogeny for many *Megabombus* species (Fig 1) in a well-supported tree. They concluded that mitochondrial 16S was the most useful gene for resolving the most recent groups within subgenera. Unfortunately, their tree has too few samples per species (usually just one) to assess the status of many Asian taxa near the rank of species and many of these Asian taxa were not included in their analysis.

A more recent study [18] includes some of the other Asian taxa and has a slightly larger sample size. This also found that another mitochondrial gene, COI, is useful at this level of analysis, because it mutates even faster than 16S. The study concluded that there are more species present in Asia, although this sample still covers only a small part of the subgenus *Megabombus*.

Above all, much of the uncertainty in the number of species in these and other earlier studies comes from using indirect criteria for recognising species: either morphology alone [14], or crude genetic divergence thresholds [11,18], both of which are now considered inappropriate [19,20,21]. In contrast, the coalescent approach relates directly to species concepts [22], for example through the application of general mixed Yule/coalescent (GMYC) models [23]. We apply GMYC models here to a much larger sample of *Megabombus* than that has been studied previously, especially with more samples from China, where these bees are most variable. We include almost all of the species of the subgenus *Megabombus* that have been accepted in recent publications [10,11,18,24,25].

To explore the evolution of long-tongued bumblebees of the subgenus *Megabombus*, we seek: (1) to recognise species; (2) to estimate dates for major events in their phylogeny; (3) to map their coarse-scale diversity; and (4) to begin to compare food-plant specialisation among the species in China.

## Materials and Methods

### Sampling bees

Specimens were collected across China between June and September 2005–2013 and deposited in the collection of the Chinese Academy of Agricultural Sciences, Institute of Apicultural Research, Beijing (IARB). Samples were identified to *Megabombus* using the keys by Williams, et al. (2008) [10]. Sample-site information was collected with a hand-held GPS (Garmin 60CS, China) and specimens were given individual identifier numbers and databased.

### DNA data

We use the single mitochondrial COI gene because earlier studies of the subgenus *Megabombus* [11,18] confirmed that mitochondrial genes have a fast mutation rate, making them more informative in analyses at the within-subgenus level. In contrast, the slower nuclear genes, such as ArgK, PEPCK, and EF-1α, are relatively uninformative at this level, so that including them would contribute little.

Genomic DNA extraction followed the protocol in our earlier publication [17]. The standard insect COI barcode region was amplified using the primers LepF1 and LepR1 [26]. Positive PCR products were sequenced from both ends by a commercial company (Biomed,Beijing, LTD) to ensure standardisation. Accession numbers for sequence data including sample IDs from the GenBank or BOLD databases (S1 Table).

### Phylogenetic analysis

ClustalX2 (version 2.0) was used for multiple alignment of sequences [27] and Collapse (version 1.2) was used to identify unique haplotypes. The best nucleotide substitution model according to jModeltest (version 2.1.3, accessed 2014) Akaike Information Criterion (AIC) [28] was GTR+I+G. Species from the subgenera *Subterranneobombus* and *Thracobombus* were used as out-groups, according to the estimate of phylogeny for *Bombus* by Cameron et al. [11]. Phylogeny was estimated with BEAST (www.beast.bio.ed.ac.uk, accessed 2014) [29], using a speciation model of a constant-size coalescent process, consistent with the null hypothesis that there is a single species. The clock model was set to lognormal relaxed clock (uncorrelated), and chain length was set to 500 million generations with a sampling frequency of one in 50,000. The consensus tree was built by TreeAnnotator (version 1.7.5, accessed 2014) with a burn-in of 1000 samples.

### Recognising species

The general mixed Yule–coalescent (GMYC) method can be used to recognize species from a single locus for insects [30]. It has been demonstrated to be an effective method for recognising even undescribed species [31,32] and has been used previously with bumblebees [33]. GMYC models were fitted with SPLITS R-package (r-forge.r-project.org/R/? group_id = 333, accessed 2013). The single threshold was used to identify a transition from intraspecific to interspecific species branching.

### Dating phylogeny

No fossils of species from this subgenus are available for dating the tree. The only available estimate is from a molecular study [34]. For the species discovered with the GMYC models, we built a tree as the best estimate of species’ phylogeny using a birth-death model for the speciation process. Events in this tree were dated with Figtree (version 1.4.2) calibrated with the molecular estimate [35].

### Mapping Diversity

**Fig 2:**
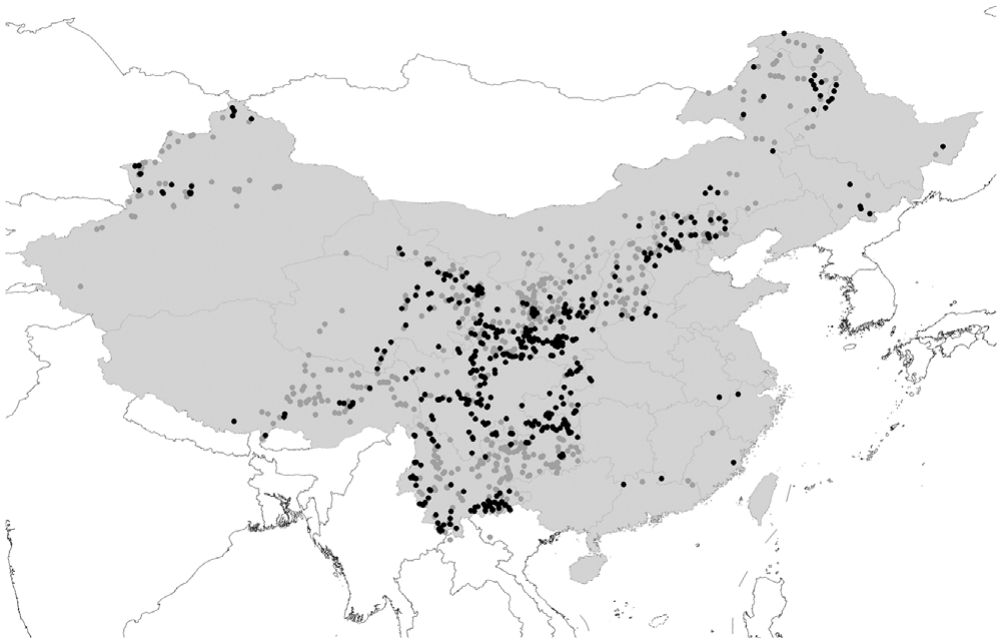
Map of sites sampled across China for bumblebees. Grey spots, all bumblebee records; black spots, records of species of the subgenus *Megabombus*. Map was created using a free computer program DIVA-GIS(http://www.diva-gis.org/download) and free spatial data (http://www.diva-gis.org/Data).

For mapping species diversity, we use an equal-area grid (Fig 2), because otherwise diversity measures are strongly affected by the size of the areas being surveyed and compared [35].

### Comparing food-plant specialisation

Data on food plants for the Chinese *Megabombus* species are available only for Sichuan and for North China [14,36]. Visits to flowers for nectar or pollen are not differentiated. Some plants are identified only to genus, although species-level identification is provided for the larger genera, such as *Pedicularis*. Plant-visit records are not associated with particular bumblebee records, so we cannot use random re-sampling of the data to compare food-plant diversity among bumblebee species for standardised sample sizes. Therefore we use a simple graphical approach, also based on the idea of species-accumulation plots. In this case we plot the food-plant diversity recorded for a bumblebee species against the total number of records for each bumblebee species, including only those bumblebee species for which we have >30 bumblebee records.

### Comparing growing degree days

Plant growth is strongly affected by growing degree days (GDD). The plants growing in a habitat are most likely to limit the food choices of bumblebees. To explore the effect of GDD on *Megabombus* species, GDD values for the sites with bee records were downloaded from the Atlas of the Biosphere (http://sage.wisc.edu/atlas)[37]. Variation in GDD for sites with bumblebee records was explored using analysis of variance (ANOVA). All analysis was done using the R project (version 3.1.1, http://www.r-project.org/).

## Results

### Recognising species

**Fig 3:**
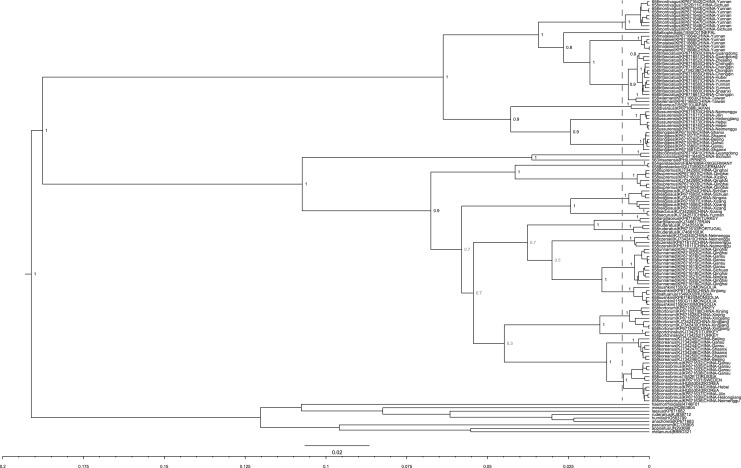
GMYC analysis to recognise species of *Megabombus*. Values at the nodes are Bayesian posterior probabilities for groups (values <0.8 are shown in grey). The scale bar represents 0.02 substitutions per nucleotide site. The single threshold of GMYC model result is shown by the vertical grey bar. Each tip is labelled with: the length of COI barcode sample sequence; the taxon name; the GenBank or BOLD ID; the sample COUNTRY and for larger countries, province.

Our COI data represent most of the Asian taxa that have been accepted recently as species. Exceptions are *B*. *senex*, from Sumatra, but unrecorded for more than 10 years, and *B*. *melanopoda*, also from Sumatra, but with only one specimen known, and unrecorded for more than a century [13]. No recent material could be sequenced for a taxon close to *B*. *hortorum* from Spain that may be a separate species, *B*. *reinigiellus* [16]. Our COI sequences lack indels, stop codons, and codon position 3 has a high %AT, so these sequences do not represent ‘numts’. GMYC model analysis (Fig 3) supports 22 species. We apply the oldest available names from the constituent taxa as the valid names for the species (ICZN, 1999).

### Dating phylogeny

**Fig 4:**
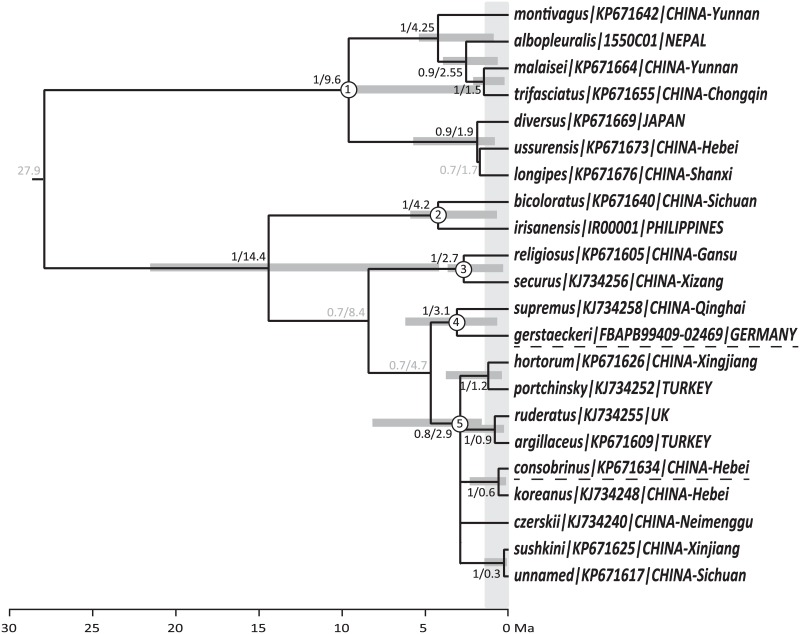
Dated estimate of phylogeny for species of the subgenus *Megabombus*. From Bayesian analysis of COI barcodes, using single samples selected to represent each of the species from Fig 2 and using the birth-death process for speciation on the tree. The tree is dated in Ma by setting the date for the divergence with the *Mendacibombus* outgroup to 34 Ma [33]. Values at each node: posterior probability / age in Ma. Nodes with support *p*≥0.8 show 95% confidence limits for the date estimate as grey bars; nodes with support *p*<0.8 have the values shown in grey; nodes with support *p*<0.66 are shown collapsed. The vertical gray line show the 1.3 Ma position to distinguish between the two time periods. The extreme food specialization species name with under dashed line.

For the species recognised in Fig 3, our best estimate for the dated species’ phylogeny is shown in Fig 4, with estimated dates and 95% interval estimates for the divergences. Two phases of *Megabombus* species radiation are recognised: (1) species in the clade node from number 1 to number 4 of Fig 4 (*B*. *montivagus* to *B*. *gerstaeckeri*) that diverged from their closest relatives in the period *ca* 4.25–1.5 Ma; and (2) species in the clade node number 5 of Fig 4 (*B*. *hortorum* to *B*. *sushkini* and ‘unnamed’) that diverged from their closest relatives’ *ca* 1.2–0.3 Ma.

### Mapping diversity

**Fig 5:**
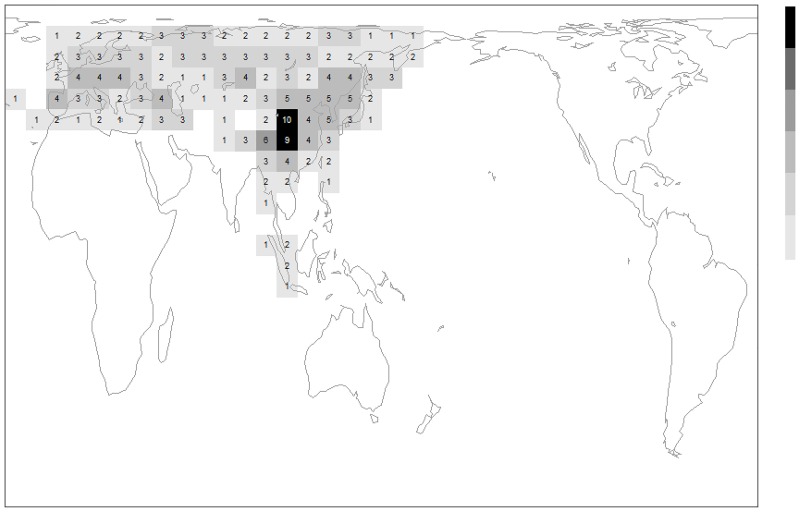
Distribution of diversity for the subgenus *Megabombus* among equal-area grid cells. Data for China are updated from the review by Williams 1998 using the IAB collection, and exclude records for known introductions (New Zealand, South America, Iceland). The grid is based on longitudinal intervals of 10°, which are used to calculate graduated latitudinal intervals to provide equal-area cells (each cell of area approximately 611,000 km²). Grey scale (right) with equal-interval richness classes. Cylindrical orthomorphic projection (excluding Antarctica) with north at the top of the map.

For the species recognised in Fig 3, we plot geographical variation in species diversity in Fig 5. Fig 5 shows that there are at least twice as many species per grid cell in cells containing the mountains on the eastern edge of the Qinghai-Tibetan plateau than there are in any more distant cells (e.g. in Europe). This is not an artefact of higher sampling effort in China, because there has been much greater sampling effort in Europe, as evidenced by the many much larger collections in national institutions in Europe (e.g. NHM, London UK).

**Fig 6:**
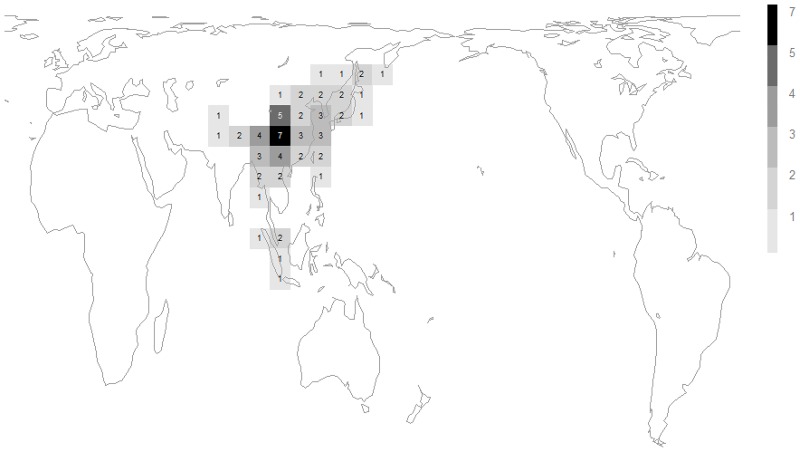
Diversity from different phases of speciation of the subgenus *Megabombus*. Data sources and grid map as in Fig 5. Richness in the species *B*. *montivagus* to *B*. *securus* from Fig 4.

**Fig 7:**
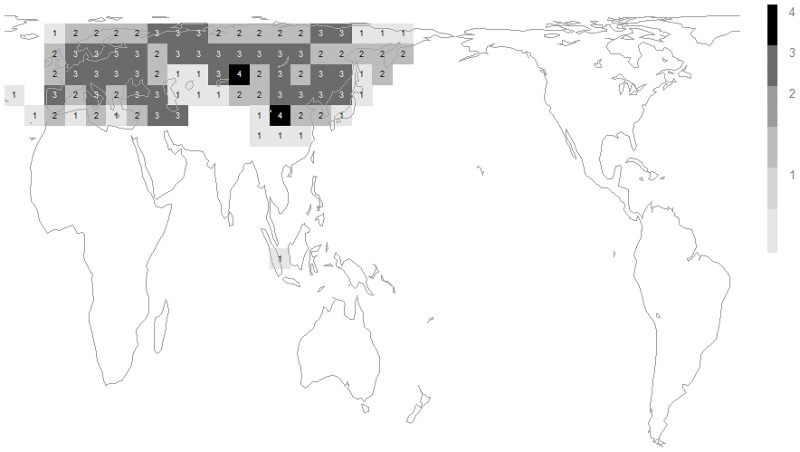
Diversity from different phases of speciation of the subgenus *Megabombus*. Data sources and grid map as in Fig 5. Richness in the species *B*. *hortorum* to *B*. *sushkini* and ‘unnamed’ from Fig 4.

The two phases of *Megabombus* species’ radiation that can be recognised from Fig 4 have different geographical distributions: (1) species in the clade node number 1 to number 3 of Fig 4 (*B*. *montivagus* to *B*. *securus*) are mostly Oriental and concentrated around the mountains at the eastern end of the Qinghai-Tibetan plateau (Fig 6); and (2) species in the clade node number 5 of Fig 4 (*B*. *hortorum* to *B*. *sushkini* and ‘unnamed’) are mostly Palaearctic and concentrated around the northern forests (Fig 7)[38].

### Comparing food-plant specialisation

**Fig 8:**
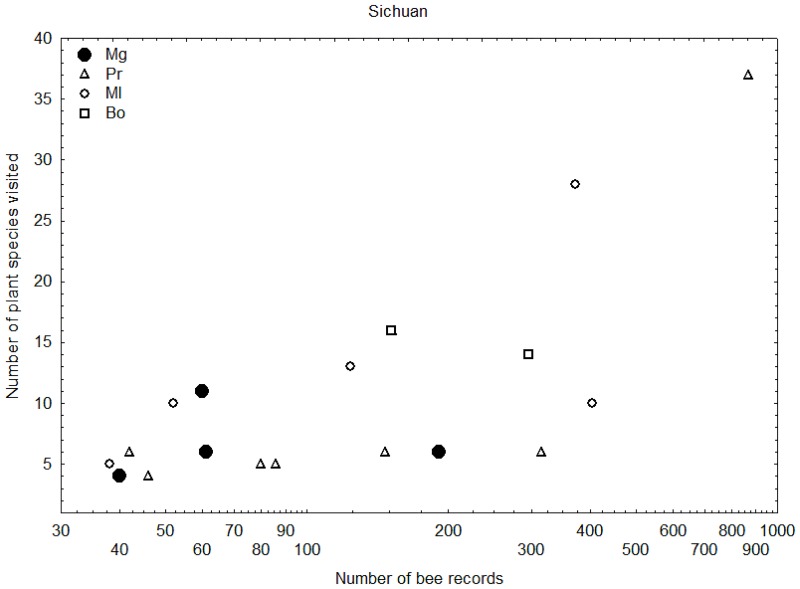
Relationship of number of food-plant species recorded per bumblebee species to number of bee records per bumblebee species. For species of the subgenera *Megabombus* (black spots), *Pyrobombus* (triangles), *Melanobombus* (circles), and *Bombus s*. *str*. (squares). Species with few food-plant-species records relative to the number of bee records are interpreted as more specialised in their fewer food-plant choices (Data from Sichuan [13]).

**Fig 9:**
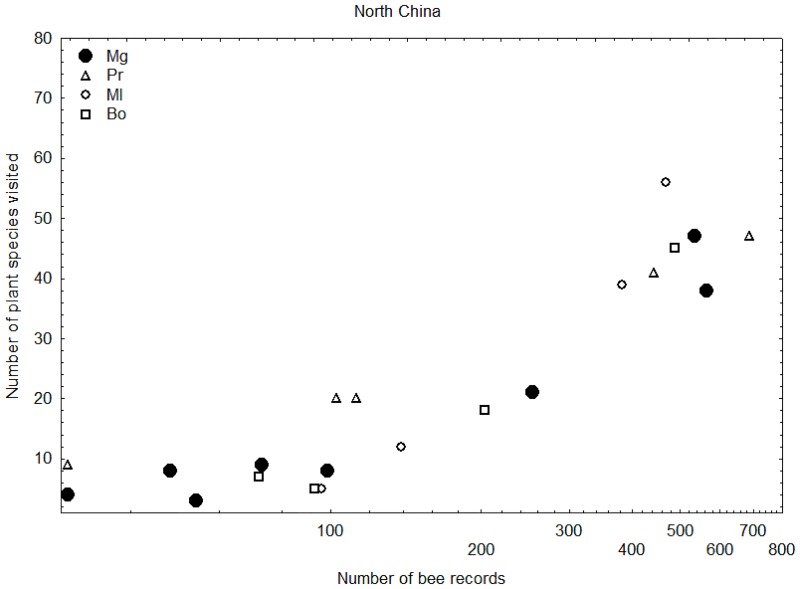
Relationship of number of food-plant species recorded per bumblebee species to number of bee records per bumblebee species. For species of the subgenera *Megabombus* (black spots), *Pyrobombus* (triangles), *Melanobombus* (circles), and *Bombus s*. *str*. (squares). Species with few food-plant-species records relative to the number of bee records are interpreted as more specialised in their fewer food-plant choices (Data from North China [14]).

Figs 8 and 9 show that Chinese *Megabombus* species tend to fall towards the lower end of the range of numbers of plant species visited for particular numbers of bumblebee records compared to the other groups of bumblebees. This provides some support for the idea that Chinese *Megabombus* species are greater food-plant specialists compared to Chinese shorter-tongued species of the subgenera *Pyrobombus*, *Melanobombus*, and *Bombus s*. *str*. in the same region.

Furthermore, these data show no support for any *Megabombus* species in Sichuan or in North China being an extreme specialist that visits just one food-plant species. However, this may have been obscured in these data because the data do not discriminate between nectar-collecting and pollen-collecting visits.

### Comparing growing degree days

**Fig 10:**
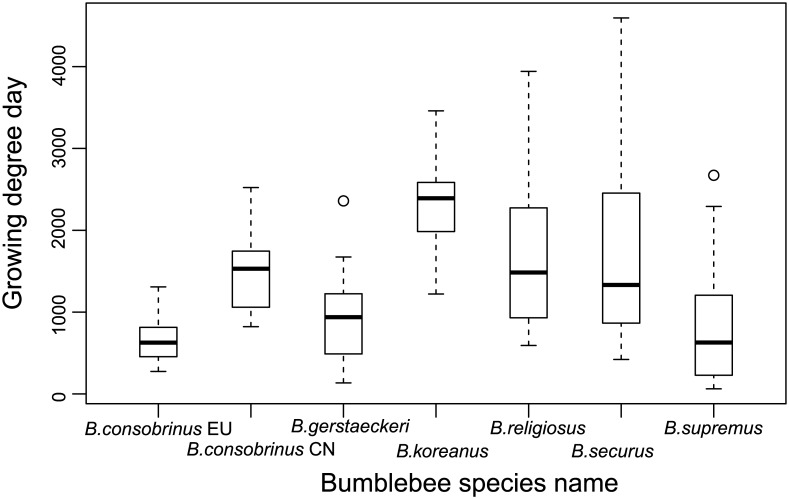
Growing degree days of collecting site for six *Megabombus* species (Boxplots show the median, upper and lower quartiles, 99% confidence limits and outliers).

The growing degree days varied not only between species but also within species (Fig 10). Global ANOVA analysis showed a significant effect of GDD among different species (F = 19.9, P<0.01). The mean value of GDD for *B*. *consobrinus*-EU + *B*. *gerstaeckeri* was significantly lower than for *B*. *religiosus* + *B*. *securus* + *B*. *koreanus* (P<0.01). Within species, *B*. *consobrinus*-EU was also significantly lower (P<0.01) compared to *B*. *consobrinus*-CN. However, *B*. *supremus* was not significantly different in GDD value compared to *B*. *consobrinus*-EU + *B*. *gerstaeckeri* (P>0.05). It is significantly lower than *B*. *religiosus* + *B*. *securus* + *B*. *koreanus* (P<0.01).

## Discussion

### Recognising species

Our analysis is not ideal because it uses only a single gene marker (part of the COI gene) and single gene trees may not always map precisely onto trees for species [23,32,39]. However, comparably fast nuclear genes could not be obtained from large samples of many of the rare mountain bees for this study because of limitations of time and budget. The results we have from other genes available from the Cameron *et al*. [11] study of smaller numbers of specimens and taxa give results that are largely compatible (Fig 1). We will discuss results for individual species in a more detailed taxonomic paper, but there are a couple of larger differences between our results and their tree.

First, our results show greater resolution with separation of the *trifasciatus-* and *diversus*-groups. The separation of these groups is also supported by characters of the morphology of the male genitalia [40].

Second, the relationships of the *religiosus*-group differs between earlier results (Fig 1) [11] and our results (Fig 4). We would expect a priori that the Cameron *et al*. [11] result would be more reliable, because it is based on more genes with its reported stronger support values. But intriguingly, our relationship for the remaining *supremus-sushkini*-group is also supported by characters of the morphology of the male genitalia (see photos in [10]).

Our analysis does have several important advantages over previous studies of this subgenus: (1) GMYC models relate directly to species concepts, in preference to previous use of divergence thresholds, which relate to species concepts only indirectly; (2) COI is a fast-evolving gene and so it is especially well suited to estimating close relationships near the species rank; (3) we have much larger samples available from which to assess population variation, especially from across the most diverse region for this group, China. Cameron *et al*. used 19 specimens and 19 sequenced samples [11]; Hines & Williams used 50 specimens and 33 sequenced samples [18]; whereas we used 4149 specimens (from China alone) and 294 sequenced samples (world-wide). Nonetheless, the sampling of North Asia in our study could be improved.

### Dating diversification

Table 1 shows that the date estimates for some species’ divergences covered in both studies are broadly similar between those obtained previously [34] and those obtained here.

**Table 1:** Directly comparable divergence times for sister species.

Divergence between species	Hines, 2008 (Ma)	This study(Ma)
*supremus/gerstaeckeri*	3.9	3.1
*hortorum/portchinsky*	2.8	1.2
*consobrinus/koreanus*	1.9	0.6
*religiosus/securus*	1.2	2.7
*ruderatus/argillaceus*	1.2	0.9

The first phase of diversification among *Megabombus* species is associated geographically with the mountains near the eastern end of the Qinghai-Tibetan plateau (Fig 6). Temporally this phase of diversification (*ca* 4.25–1.5 Ma) is associated with the late rise of this eastern end of the plateau in the Hengduan mountain system [41]. Presumably the late rise of these mountains created both many new barriers and many new opportunities by fragmenting the mountain forest habitat for *Megabombus* species.

The second phase of diversification among *Megabombus* species is associated geographically with the northern (boreal) forests (Fig 7)[38]. Temporally this phase of diversification (1.2–0.3 Ma) is associated with the Plio-Pleistocene cooling in global climate and onset of the ice ages [42,43]. Presumably the onset of the glaciations again created both many new barriers and many new opportunities by fragmenting the northern forest habitat for *Megabombus* species.

### Evolution of food-plant specialisation

As far as we can tell from available data, extreme food-plant specialisation has evolved just twice in the subgenus *Megabombus*: in *B*. *gerstaeckeri* [44,45] and in *B*. *consobrinus* [46,47,48]. Curiously, the species with the most extreme long tongues, including *B*. *religiosus*, *B*. *securus*, and *B*. *supremus*, have multiple food-plant species in Sichuan and do not appear to be among the most extreme food plant specialists [14]. Extreme specialisation is no doubt related to the abundance of a suitable food-plant species. But it has also been suggested that extreme specialisation is likely to be advantageous for social species like bumblebees only if they have small colonies and live in extreme habitats that have short foraging seasons, so that a long succession of flowering by different food-plant species through the summer is not required for colony reproductive success [49]. Both of the extreme specialist species (*B*. *gerstaeckeri* and *B*. *consobrinus*) occur in habitats with short foraging seasons, in subalpine and subarctic meadows respectively. In both cases their sister species are not extreme food-plant specialists (*B*. *supremus* [14,36]; *B*. *koreanus* [50]), even though *B*. *supremus* also occurs at high elevation with a low GDD value (3529–4464 m in Sichuan [14]). It was reported that the change of food plant of *B*. *supremus* was affected heavily by the reductions of flowers in the food plants *Hedysarum* and *Saussurea*[51]. This may have led to a temporary increase in the diversity food plants used by the declining *B*. *supremus*, a situation that may not be stable in the long term.

In the east, one of the most specialised species (*B*. *consobrinus*) may have spread south from Russia into North China, where it has become less specialised in its food-plant choices [36] than in Europe. This remarkable exception might be a response to the longer foraging season further south, demonstrating the importance of this factor. But detailed comparative studies of *Megabombus* foraging activity are now needed.

## Supporting Information

S1 File Detailed results of GMYC species delimitation base on the Concatenated ultrametric tree. (DOCX)

S2 File Plot of (y axis) the log likelihood of the single threshold GMYC model for the Bayesian tree of unique COI-barcode haplotypes against (x axis) substitutions per nucleotide site. (PDF)

S3 File Threshold from the GMYC model at maximum likelihood was also showed. (PDF)

S4 File Letter of Authorization from Institute of Apicultural Reasearch, Chinese Academy of Agricultural Sciences. (PDF)

S1 Table Collection localities, sample deposition and GenBank accession numbers for specimens used in molecular analyses. Asterisks for sequence ID are deposited in BOLD.
(DOCX)
