# Supplementary material for: Extreme Food-Plant Specialisation in Megabombus Bumblebees as a Product of Long Tongues Combined with Short Nesting Seasons
Source: PLoS One. 2015 Aug 12;10(8):e0132358. doi: 10.1371/journal.pone.0132358 (PMC4534414; doi:10.1371/journal.pone.0132358)
Supplement: S1 File — (DOCX) [file pone.0132358.s001.docx]

**Result of GMYC species delimitation base on the Concatenated ultrametric tree:**

**Method: single**

Likelihood of null model: 943.0329

Maximum likelihood of GMYC model: 965.5214

Llikelihood ratio: 44.97695

Result of LR test: 1.711511e-10***

Number of ML clusters: 20

Confidence interval: 20-24

Number of ML entities: 31

Confidence interval: 31-38

Threshold time: -0.007934946

**Method: multiple**

Likelihood of null model: 943.0329

Maximum likelihood of GMYC model: 967.9219

Likelihood ratio: 49.77789

Result of LR test: 1.551914e-11***

Number of ML clusters: 25

Confidence interval: 19-25

Number of ML entities: 38

Confidence interval: 32-45

Threshold time: -0.007934946

-0.004325968

-0.002794367

-0.00251208
