# Supplementary figures and images for: Extreme Food-Plant Specialisation in Megabombus Bumblebees as a Product of Long Tongues Combined with Short Nesting Seasons
Source: PLoS One. 2015 Aug 12;10(8):e0132358. doi: 10.1371/journal.pone.0132358 (PMC4534414; doi:10.1371/journal.pone.0132358)

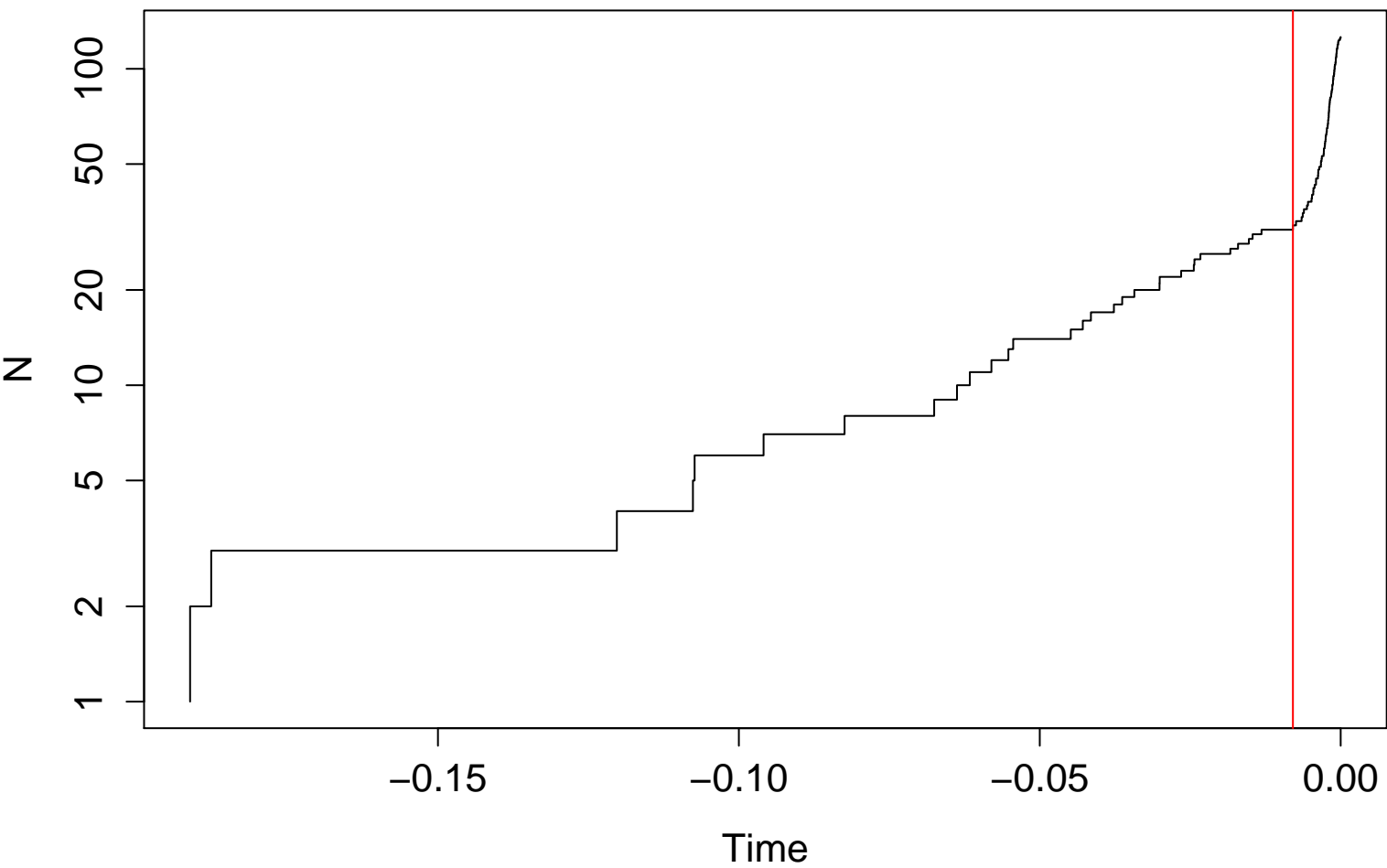

Supplement: S2 File — (PDF) [file pone.0132358.s002.pdf]

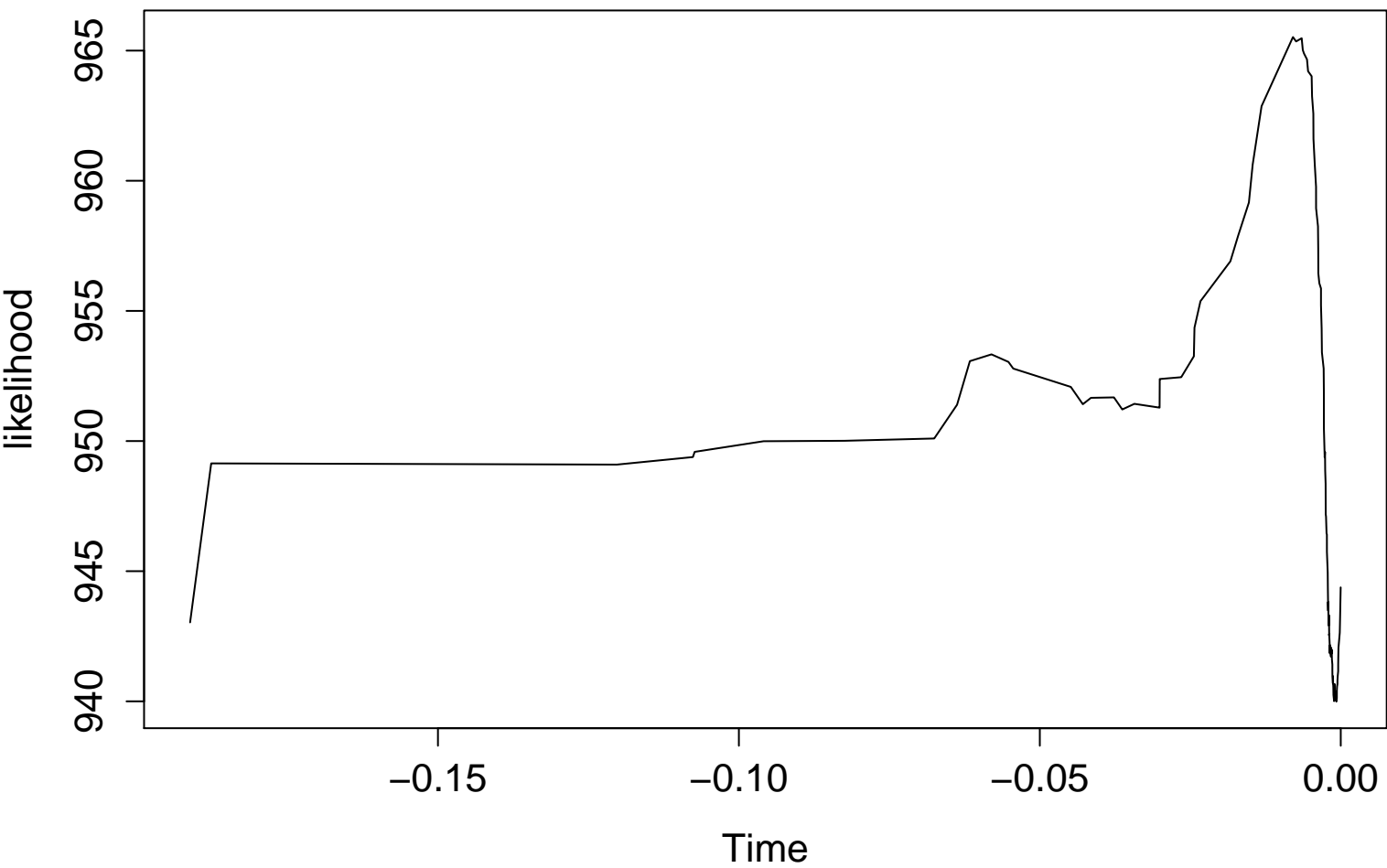

Supplement: S3 File — (PDF) [file pone.0132358.s003.pdf]
