# Supplementary material for: Extreme Food-Plant Specialisation in Megabombus Bumblebees as a Product of Long Tongues Combined with Short Nesting Seasons
Source: PLoS One. 2015 Aug 12;10(8):e0132358. doi: 10.1371/journal.pone.0132358 (PMC4534414; doi:10.1371/journal.pone.0132358)
Supplement: S4 File — (PDF) [file pone.0132358.s004.pdf]

Letter of Authorization

Manuscript by Huang Jiaxing et al.: *Extreme food-plant specialisation in Megabombus bumblebees as a product of long tongues combined with short nesting seasons*

Dear Sir / Madam,

We at the Institute of Apiculture, Chinese Academy of Agricultural Sciences, guarantee that all of the specimens used in the preparation of the above manuscript were collected legally as authorized by the appropriate administrative departments. All of the specimens were collected as part of projects administered by the China Agriculture Research System (CARS-45) and the Special Fund for Agro-scientific Research in the Public Interest (201203080-4).

Please give details of any further authorization required for publication, in particular of any form of words that would need to be added as a statement in the manuscript. Your assistance in this would be much appreciated.

Sincerely yours,

中国农业科学院蜜蜂研究所

Director
Prof. Peng Wenjun
Institute of Apiculture
Chinese Academy of Agricultural Sciences
Phone:+8610-62597059
