# Supplementary material for: Extreme Food-Plant Specialisation in Megabombus Bumblebees as a Product of Long Tongues Combined with Short Nesting Seasons
Source: PLoS One. 2015 Aug 12;10(8):e0132358. doi: 10.1371/journal.pone.0132358 (PMC4534414; doi:10.1371/journal.pone.0132358)
Supplement: S1 Table — Asterisks for sequence ID are deposited in BOLD. (DOCX) [file pone.0132358.s005.docx]

**S1 Table**

**Collection localities, sample deposition and GenBank accession numbers for specimens used in molecular analyses.**

| Taxon | Locality | Collector | Latitude | Longitude | depository | Genbank ID |
| --- | --- | --- | --- | --- | --- | --- |
| gerstaeckeri | DE, Schachen GAP | J. Voith | N 47.572 | E 11.128 | BSCZ | KT334300 |
| gerstaeckeri | SE, Loetschenpass | C. Schmid-Egger | N 46.4 | E 7.7 | BSCZ | HM401268 |
| supremus | CN, Dachamuchang | J. Huang | N 38.69 | E 99.52 | IAB | KJ734258 |
| supremus | CN, Xiega | Z. Miao | N 31.97 | E 96.32 | IAB | KJ734259 |
| supremus | CN, Jielongxiang | Z. Miao | N 33.20 | E 96.39 | IAB | KP671601 |
| supremus | CN, Pali | S. Luo | N 27.70 | E 89.14 | IAB | KP671602 |
| supremus | CN, Dahebai | Z. Miao | N 35.88 | E 99.69 | IAB | KP671603 |
| supremus | CN, Dahebai | Z. Miao | N 35.88 | E 99.69 | IAB | KP671604 |
| religious | CN, Yehegu | S. Luo | N 35.51 | E 106.23 | IAB | KJ734253 |
| religious | CN, Leidongping | X. Zhang | N 29.50 | E 103.33 | IAB | KJ734254 |
| religious | CN, Leidongping | X. Zhang | N 29.50 | E 103.33 | IAB | KP671605 |
| religious | CN, Namulin | S. Luo | N 29.70 | E 94.73 | IAB | KP671606 |
| religious | CN, Dazhoushao | S. Luo | N 29.57 | E 94.57 | IAB | KP671607 |
| religious | CN, Baibazhen | S. Luo | N 29.79 | E 93.89 | IAB | KP671608 |
| securus | CN, Dingri | Z. Miao | N 28.61 | E 87.13 | IAB | KJ734256 |
| securus | CN, Xiaozhongdian | C. Liang | N 21.45 | E 99.82 | IAB | KJ734257 |
| argillaceus | IR, Werk | A. Monfared | N 36.454 | E 50.687 | NHM | KJ746617 |
| argillaceus | TR, Artvin | M. Aytekin | N 41.076 | E 42.208 | IAB | KP671609 |
| ruderatus | UK, Kent | P. Williams | N 51.35 | E 1.43 | IAB | KJ734255 |
| ruderatus | UK, Shoreham | J. Huang | N 51.334 | E 0.178 | IAB | KJ746616 |
| ruderatus | UK, Santana | Lakache | N 37.85 | W 25.26 | NHM | KP671610 |
| czerskii | CN, Majiazi | J. Huang | N 43.22 | E 117.32 | IAB | KJ734240 |
| czerskii | CN,Majiazi | J. Huang | N 43.22 | E 117.32 | IAB | KJ734241 |
| czerskii | CN, Qijiagou | J. Huang | N 45.92 | E 121.23 | IAB | KP671611 |
| czerskii | CN, Xintubu | J. Huang | N 48.27 | E 119.75 | IAB | KP671612 |
| unnamed | CN, Roadside | S. Luo | N 37.08 | E 103.01 | IAB | KP671613 |
| unnamed | CN, Tiejingshan | J. Huang | N 39.31 | E 97.91 | IAB | KP671614 |
| unnamed | CN, Roadside | J. Huang | N 39.69 | E 97.73 | IAB | KP671615 |
| unnamed | CN, Huangcheng | A. Han | N 37.88 | E 101.76 | IAB | KP671616 |
| unnamed | CN, Banyou | Z. Zhou | N 37.88 | E 101.76 | IAB | KP671617 |
| unnamed | CN, Guangjiaonihe | S. Zhang | N 37.19 | E 98.86 | IAB | KP671618 |
| unnamed | CN, Guangjiaonihe | S. Zhang | N 37.19 | E 98.86 | IAB | KP671619 |
| unnamed | CN, Heisi | J. Huang | N 36.34 | E 98.25 | IAB | KP671620 |
| unnamed | CN, Yueliangshan | B. Wang | N 36.20 | E 105.62 | IAB | KP671621 |
| unnamed | CN, Waligeituo | Z. Miao | N 33.10 | E 96.78 | IAB | KP671622 |
| unnamed | CN, Ejia | Z. Miao | N 36.20 | E 101.56 | IAB | KP671623 |
| sushkini | MN, Hovsgol Nuur | D. Sheppard | N 51.05 | E 100.733 | P. Williams | GU674510 |
| sushkini | MN, Hovsgol Nuur | D. Sheppard | N 51.05 | E 100.733 | P. Williams | GU674515 |
| sushkini | MN, Hovsgol Nuur | D. Sheppard | N 51.05 | E 100.733 | P. Williams | KT334301 |
| sushkini | MN, Hovsgol Nuur | D. Sheppard | N 51.05 | E 100.733 | P. Williams | KT334302 |
| sushkini | MN, Hovsgol Nuur | D. Sheppard | N 51.05 | E 100.733 | P. Williams | KP671624 |
| sushkini | CN, Xiaodonggou | J. Huang | N 47.99 | E 88.24 | IAB | KP671625 |
| hortorum | CN, Nalati | Z. Zhou | N 43.19 | E 84.34 | IAB | KJ734242 |
| hortorum | CN, Guozigou | Z. Zhou | N 44.46 | E 81.11 | IAB | KJ734243 |
| hortorum | CN, Guozigou | Z. Zhou | N 44.46 | E 81.11 | IAB | KP671626 |
| hortorum | CN, Xiaodonggou | J. Huang | N 47.99 | E 88.24 | IAB | KP671627 |
| hortorum | CN, Xiaodonggou | J. Huang | N 47.99 | E 88.24 | IAB | KP671628 |
| hortorum | CN, Yamate | J. An | N 44.96 | E 80.80 | IAB | KP671629 |
| hortorum | TY, Artvin | M. Aytekin | N 41.076 | E 42.208 | IAB | KP671630 |
| portchinsky | TY, Kars | P. Rasmont | - | - | P. Rasmont | KJ734251 |
| portchinsky | TY, Artvin | M. Aytekin | N 41.076 | E 42.208 | IAB | KJ734252 |
| koreanus | CN, Chengxian | J. Huang | N 33.72 | E 105.61 | IAB | KJ734244 |
| koreanus | CN, Xiaolongmen | J. Huang | N 39.97 | E 115.43 | IAB | KJ734245 |
| koreanus | CN, Baihuashan | J. Huang | N 40.53 | E 115.75 | IAB | KJ734246 |
| koreanus | CN, Ciba | J. An | N 33.8 | E 106.63 | IAB | KJ734247 |
| koreanus | CN, Ciba | J. An | N 33.8 | E 106.63 | IAB | KJ734248 |
| koreanus | CN, Shimen | Z. Miao | N 34.41 | E 106.11 | IAB | KJ734249 |
| koreanus | CN, Liuba | Y. Zhang | N 33.78 | E 107.18 | IAB | KJ734250 |
| consobrinus | KR, Nodong-li | Tripotin | N 37.7013 | E 128.482 | P. Rasmont | HQ553043 |
| consobrinus | KR, Nodong-li | Tripotin | N 37.7013 | E 128.482 | P. Rasmont | HQ553042 |
| consobrinus | RS, Vyskoye village | D. Bennett | N 46.8063 | E 142.337 | U Kansas | KT334303 |
| consobrinus | SW, Ramundberget | P. Williams | N 62.70 | E 12.78 | NHM | KP671631 |
| consobrinus | CN, Dianzixiang | J. Huang | N 34.61 | E 103.71 | IAB | KP671632 |
| consobrinus | CN, Dianzixiang | J. Huang | N 34.61 | E 103.71 | IAB | KP671633 |
| consobrinus | CN, Wulingshan | J. Huang | N 40.60 | E 117.48 | IAB | KP671634 |
| consobrinus | CN, Amuquhu | J. Huang | N 34.89 | E 102.65 | IAB | KP671635 |
| consobrinus | CN, Shierligou | J. Li | N 49.42 | E 121.05 | IAB | KP671636 |
| consobrinus | CN, Duizigou | J. An | N 41.92 | E 127.85 | IAB | KP671637 |
| consobrinus | CN, Zhagana | J. An | N 34.23 | E 103.17 | IAB | KP671638 |
| consobrinus | CN, Tahe | Z. Zhou | N 52.35 | E 124.71 | IAB | KP671639 |
| irisanensis | PH, Cambulo | N. Layron | N 16.952 | E 121.128 | University of Vienna | - |
| bicoloratus | CN, Roadside | J. Li | N 28.76 | E 102.59 | IAB | KP671640 |
| bicoloratus | CN, Lianshan | - | N 24.57 | E 112.08 | IAB | KP671641 |
| montivagus | CN, Daweishan | C. Liang | N 22.96 | E 103.69 | IAB | KP671642 |
| montivagus | CN, ChangChiaTsun | P. Williams | N 26.58 | E 102.37 | Paul Williams | KT334304 |
| montivagus | CN, Daweishanzhongduan | W. Luo | N 22.98 | E 103.68 | IAB | KP671643 |
| montivagus | CN, Laozhi | W. Luo | N 23.32 | E 103.54 | IAB | KP671644 |
| montivagus | CN, Xiaomengyang | Y. Zhao | N 22.02 | E 100.96 | IAB | KP671645 |
| montivagus | CN, Galenong | Y. Zhao | N 21.57 | E 100.35 | IAB | KP671646 |
| montivagus | CN, Qilubai | C. Liang | N 23.20 | E 103.51 | IAB | KP671647 |
| montivagus | CN, Mengshanaguai | C. Liang | N 23.70 | E 99.58 | IAB | KP671648 |
| montivagus | CN, ChangChiaTsun | P. Williams | N 26.58 | E 102.37 | Paul Williams | KP671649 |
| albopleuralis | NEP, Khari Lagna | M. Hartmann | N 29.369 | E 82.153 | NKM Erfurt | KT334305 |
| trifasciatus | CN, Xinglong cun | J. Guo | N 28.91 | E 107.27 | IAB | KJ734238 |
| trifasciatus | CN, Chebaling | J. An | N 24.94 | E 114.19 | IAB | KP671650 |
| trifasciatus | CN, Chebaling | J. An | N 24.94 | E 114.19 | IAB | KP671651 |
| trifasciatus | CN, Xiaohechang | P. Williams | N 29.82 | E 102.76 | P. Williams | KP671652 |
| trifasciatus | CN, Laoshimianshan | J. Guo | N 28.60 | E 106.40 | IAB | KP671653 |
| trifasciatus | CN, Xinglong cun | J. Guo | N 28.91 | E 107.27 | IAB | KP671654 |
| trifasciatus | CN, Xinglong cun | J. Guo | N 28.91 | E 107.27 | IAB | KP671655 |
| trifasciatus | CN, Xujiazhuang | J. Yao | N 39.69 | E 117.51 | IAB | KP671656 |
| trifasciatus | CN, Shaozipo | D. Song | N 39.69 | E 117.51 | IAB | KP671657 |
| trifasciatus | CN, Shaozipo | D. Song | N 39.69 | E 117.51 | IAB | KP671658 |
| trifasciatus | CN, Changanxiang | D. Song | N 27.83 | E 104.83 | IAB | KP671659 |
| trifasciatus | CN, Changqing | Y. Zhang | N 33.59 | E 107.51 | IAB | KP671660 |
| trifasciatus | CN, Dabanying | J. Guo | N 29.29 | E 108.99 | IAB | KP671661 |
| wilemani | CN, Nantou meifeng | C. Lin | - | - | C. Starr | KP671662 |
| wilemani | CN, Nantou meifeng | C. Lin | - | - | C. Starr | KP671663 |
| malaisei | CN, Daweishan | C. Liang | N 22.95 | E 103.70 | IAB | KP671664 |
| malaisei | CN, Cikai | C. Liang | N 27.73 | E 98.68 | IAB | KP671665 |
| malaisei | CN, Bashao | C. Liang | N 25.98 | E 98. 80 | IAB | KP671666 |
| malaisei | CN, Daweishanding | W. Luo | N 22.90 | E 103.68 | IAB | KP671667 |
| malaisei | CN, Daweishanding | W. Luo | N 22.90 | E 103.68 | IAB | KP671668 |
| diversus | RU, S Okhoskoye town | T. Anderson | - | - | U Kansas | KT334306 |
| diversus | JP, Hanliang | H. Xu | - | - | IAB | KP671669 |
| ussurensis | CN, Ganhe | Z. Zhou | N 22.90 | E 103.68 | IAB | KP671670 |
| ussurensis | CN, Fengman | J. Huang | N 43.79 | E 126.57 | IAB | KP671671 |
| ussurensis | CN, Panlongshan | Z. Zhou | N 50.15 | E 125.70 | IAB | KP671672 |
| ussurensis | CN, Fengning | J. Huang | N 41.36 | E 116.62 | IAB | KP671673 |
| ussurensis | CN, Fengning | J. Huang | N 41.36 | E 116.62 | IAB | KP671674 |
| ussurensis | CN, Laolai | Z. Zhou | N 48.69 | E 125.00 | IAB | KP671675 |
| longipes | CN, Wangfengshi | J. An | N 39.25 | E 114.35 | IAB | KP671676 |
| longipes | CN, Dukang | X. Liu | N 36.02 | E 109.45 | IAB | KP671677 |
| longipes | CN, Panpo | S. Luo | N 37.66 | E 101.32 | IAB | KP671678 |
| longipes | CN, Liziba | Z. Zhou | N 32.91 | E 104.36 | IAB | KP671679 |
| longipes | CN, Lazikou | J. An | N 33.10 | E 103.93 | IAB | KP671680 |
| longipes | CN, Diaoyutai | Y. Zhang | N 33.83 | E 107.77 | IAB | KP671681 |
| haemorrhoidalis | TH, Doi Phu Kha NP | Charoen & Nikom | N 19.2022 | E 101.079 | L. Packer | KT334307 |
| mesomelas | IT, Gran Sasso | C. Schmid-Egger | N 42.469 | E 13.564 | BSCZ | HQ563803 |
| laesus | CN, Guanyindian | J. Huang | N 40.22 | E 115.15 | IAB | KP671682 |
| ruderarius | DE, Nuernberg | M. Kraus | N 49.45 | E 11.067 | BSCZ | GU705935 |
| humilis | DE, Passau | C. Schmid-Egger | N 48.572 | E 13.59 | BSCZ | HQ563799 |
| anachoreta | CN, Baoqing | J. Huang | N 46.21 | E 132.53 | IAB | KP671683 |
| pascuorum | KP, South Korea | C. Jung | N 35.594 | E 128.245 | - | KC135905 |
| appositus | CA, Kootenay NP | BIObus 2010 | N 50.675 | W 115.931 | BBCHY | JN293705 |
| melanurus | CN, Shuangjingdian | P. Williams | N 43.247 | E 118.108 | P. Williams | KT334308 |

IAB: Institute of Apiculture, Chinese Academy of Agricultural Sciences, Beijing, China; NHM, Natural History Museum, London, UK; BSCZ , Bavarian State Collection of Zoology, Germany. BBCHY, Biodiversity Institute of Ontario, Ontario, Canada.
